# Prognostic impacts of changes in left ventricular ejection fraction in heart failure patients with preserved left ventricular ejection fraction

**DOI:** 10.1136/openhrt-2019-001112

**Published:** 2020-04-05

**Authors:** Akiomi Yoshihisa, Yu Sato, Yuki Kanno, Mai Takiguchi, Tetsuro Yokokawa, Satoshi Abe, Tomofumi Misaka, Takamasa Sato, Masayoshi Oikawa, Atsushi Kobayashi, Takayoshi Yamaki, Hiroyuki Kunii, Yasuchika Takeishi

**Affiliations:** 1 Department of Cardiovascular Medicine, Fukushima Medical University, Fukushima, Japan; 2 Department of Advanced Cardiac Therapeutics, Fukushima Medical University, Fukushima, Japan; 3 Department of Pulmonary Hypertension, Fukushima Medical University, Fukushima, Japan

**Keywords:** heart failure, heart failure with normal ejection fraction, cardiac remodelling

## Abstract

**Background:**

It has been reported that recovery of left ventricular ejection fraction (LVEF) is associated with better prognosis in heart failure (HF) patients with reduced EF (rEF). However, change of LVEF has not yet been investigated in cases of HF with preserved EF (HFpEF).

**Methods and results:**

Consecutive 1082 HFpEF patients, who had been admitted to hospital due to decompensated HF (EF >50% at the first LVEF assessment at discharge), were enrolled, and LVEF was reassessed within 6 months in the outpatient setting (second LVEF assessment). Among the HFpEF patients, LVEF of 758 patients remained above 50% (pEF group), 138 patients had LVEF of 40%–49% (midrange EF, mrEF group) and 186 patients had LVEF of less than 40% (rEF group). In the multivariable logistic regression analysis, younger age and presence of higher levels of troponin I were predictors of rEF (worsened HFpEF). In the Kaplan-Meier analysis, the cardiac event rate of the groups progressively increased from pEF, mrEF to rEF (log-rank, p<0.001), whereas all-cause mortality did not significantly differ among the groups. In the multivariable Cox proportional hazard analysis, rEF (vs pEF) was not a predictor of all-cause mortality, but an independent predictor of increased cardiac event rates (HR 1.424, 95% CI 1.020 to 1.861, p=0.039).

**Conclusion:**

An initial assessment of LVEF and LVEF changes are important for deciding treatment and predicting prognosis in HFpEF patients. In addition, several confounding factors are associated with LVEF changes in worsened HFpEF patients.

**Key questions**

**What is already known about this subject?**

- Changes in left ventricular ejection fraction (LVEF) and its prognostic impact on patients with heart failure with reduced EF (HFrEF) have recently been reported on; it has been suggested that the recovery of LVEF, known as ‘recovered EF’, occurs in a proportion of HFrEF patients, and is associated with better prognosis. However, LVEF changes, their clinical characteristics and prognostic impacts in HF patients with preserved EF (HFpEF) are unclear.

**What does this study add?**

- Of consecutive 1082 HFpEF patients, 186 (17.2%) had LVEF of less than 40% at the second LVEF assessment (worsened HFpEF). Younger age, presence of coronary artery disease and sleep-disordered breathing, higher levels of troponin I and left ventricular end diastolic dimension were predictors of worsened HFpEF, which was associated with increased cardiac event rates.

**How might this impact on clinical practice?**

- An initial assessment of LVEF and LVEF changes are important for deciding treatment and predicting prognosis in HFpEF patients. In addition, several confounding factors are associated with LVEF changes in worsened HFpEF patients.

## Introduction

Left ventricular ejection fraction (LVEF) is among the most ingrained and commonly used quantities in clinical practice. LVEF is used in the diagnosis, characterisation, prognosis, patient triage and treatment selection of heart failure (HF).^1–5^ HF with reduced EF (HFrEF; LVEF <40%) is well characterised and established for evidence-based therapy,^3–5^ whereas HF with preserved EF (HFpEF; LVEF ≥50%) is a common and complex syndrome without evidence-based therapy.^6 7^ On the other hand, changes in LVEF and its prognostic impact on HFrEF patients have recently been reported on^8^; it has been suggested that the recovery of EF, known as recovered EF, occurs in a proportion of HFrEF patients, and is associated with better prognosis.^9–14^ However, LVEF changes, their clinical characteristics and prognostic impacts in patients with HFpEF are unclear.

Therefore, the aim of the current study was to clarify LVEF changes, their clinical characteristics and prognostic impacts in patients with HFpEF.

## Methods

This was a prospective observational study of 1161 decompensated HFpEF patients, who were discharged from Fukushima Medical University Hospital between 2010 and 2016, with LVEF ≥50% at discharge. The diagnosis of decompensated HF was made by several cardiologists based on the HF guidelines.^3–5^ Patients who had been admitted due to acute coronary syndrome and/or had previously undergone haemodialysis were excluded. All patients underwent echocardiography, and LVEF was assessed and HFpEF was determined at hospital discharge (first assessment), then premeditatedly reassessed in 1082 patients in the outpatient setting within 6 months (mean 3 months, range 2–6 months) postdischarge (second assessment). Of the 1161 patients, the second assessment was not performed in 75, based on circumstances of the patients or the physicians, and four patients died or were hospitalised due to decompensated HF before the second assessment. We divided the remaining 1082 patients into three groups according to changes in LVEF observed at the second assessment: remained pEF (LVEF ≥50%, n=758); mid-range LVEF (mrEF) (LVEF 40%–49%, n=138) and reduced LVEF (rEF) (LVEF <50%, n=186).

We compared the patients’ clinical features, laboratory data, echocardiography and ECG parameters, and postdischarge prognosis. The patients were followed up until 2018 for cardiac events and all-cause death. Cardiac events were defined as worsened HF and cardiac death. Cardiac death was classified by independent experienced cardiologists as death from worsened HF, ventricular fibrillation documented by ECG or implantable devices, or acute coronary syndrome. Worsened HF was defined as hospitalisation due to decompensated HF. Postdischarge, the patients visited our hospital or their referring hospital once every 1–2 months. Status and dates of death were obtained from the patients’ medical records. If these data were unavailable, status was ascertained by a telephone call to the patient’s referring hospital physician. We were able to follow up on all patients who had undergone the second assessment. Those administering the survey were blind to the analyses, and written informed consent was obtained from all study subjects.^15^

We evaluated several comorbidities that often coexist and are associated with adverse prognosis in HF patients.^16^ Coronary artery disease (CAD) was confirmed by the following: myocardial scintigraphy, coronary CT angiography and/or coronary angiography. Atrial fibrillation (AF) was identified by ECG performed during hospitalisation and/or from medical records. Hypertension was defined as the recent use of antihypertensive drugs, systolic blood pressure ≥140 mm Hg and/or diastolic blood pressure ≥90 mm Hg. Diabetes mellitus was defined as the recent use of antidiabetic drugs, a fasting glucose value of ≥126 mg/dL, a casual glucose value of ≥200 mg/dL and/or HbA1c ≥6.5% (National Glycohemoglobin Standardization Program). Dyslipidaemia was defined as the recent use of cholesterol-lowering drugs, a triglyceride value of ≥150 mg/dL, a low-density lipoprotein cholesterol value of ≥140 mg/dL and/or a high-density lipoprotein cholesterol value of <40 mg/dL. Chronic kidney disease (CKD) was defined as an estimated glomerular filtration rate of <60 mL/min/1.73 m^2^ according to the Modification of Diet in Renal Disease formula.^17 18^ Anaemia was defined as haemoglobin levels of <12.0 g/dL in females and <13.0 g/dL in males.^5^ Hyperuricaemia was defined as regular usage of antihyperuricemic agents or serum uric acid levels of over 7 mg/dL.^19^ Sleep-disordered breathing (SDB) was defined as apnoea–hypopnoea index of >5 times/hour, and included both central and obstructive SDB, determined by a portable sleep monitor, polysomnography and/or from medical records.^20–22^ Chronic obstructive pulmonary disease was defined as forced expiratory volume in one second/forced vital capacity of <70% by spirometry according to the Global Initiative for Chronic Obstructive Lung Disease, the American Thoracic Society/European Respiratory Society guidelines, and/or from medical records.^23^ Peripheral artery disease was diagnosed according to the recent guidelines using CT, angiography and/or ankle-brachial index.^24 25^

### Measurement of parameters of laboratory data, ECG and echocardiography

Blood samples were obtained from all patients at Fukushima Medical University Hospital at hospital discharge. B-type natriuretic peptide (BNP) levels were measured using a specific immunoradiometric assay (Shionoria BNP kit, Shionogi, Osaka, Japan). High-sensitivity troponin I levels were measured using EDTA anticoagulated plasma with a refined assay (Abbott-Architect, Abbott Laboratories, Abbott Park, Illinois, USA).

The standard resting ECG was recorded in the supine position with CardioStar FCP-7541 (Fukuda Denshi, Tokyo, Japan) and stored digitally. This system allows automatic measuring of QT and QTc interval. The QT interval was measured from the beginning of the QRS complex until the T wave returned to the isoelectric line. The median QT interval was then calculated and corrected for the heart rate.^18^

Echocardiography was performed blindly by experienced echocardiographers using standard techniques.^16 23^ The echocardiographic parameters investigated included left ventricular diastolic dimension (LVDd), left ventricular systolic dimension (LVDs), LVEF, left atrium volume, ratio of early transmitral flow velocity to mitral annular velocity (mitral valve E/e’), inferior vena cava diameter (IVC), tricuspid regurgitation pressure gradient (TR-PG) and right ventricular fractional area change (RV-FAC).^26^ The LVEF was calculated using Simpson’s method in a four-chamber view.^16 23^ The intraobserver variability (the SD of the differences/average value) of LVEF was 6%±2%. The RV-FAC, defined as (end-diastolic area and end-systolic area)/end diastolic area × 100, was used as a measure of right ventricular systolic function. All measurements were performed using ultrasound systems (ACUSON Sequoia, Siemens Medical Solutions USA, Mountain View, California, USA).

### Statistical analysis

Categorical variables are expressed as numbers and percentages. A X^2^ test was used for comparisons of categorical variables, followed by Fisher’s exact test when appropriate. Normality was confirmed using the Shapiro-Wilk test in each group. Parametric variables are presented as mean±SD and non-parametric variables (eg, BNP, troponin I and C reactive protein) are presented as a median and IQR. Parametric variables were compared using analysis of variance (ANOVA), and equality was tested using the Levene test. If the data were equal, ANOVA was followed by Tukey’s honest significant difference. If the data were not equal, the Games-Howell post hoc test was used. Non-parametric variables were compared using the Kruskal-Wallis test. We performed logistic regression analysis allowing for interaction between the onset of rEF; worsened HFpEF and each possible confounding factor. Kaplan-Meier analysis was used for presenting the cardiac event rate and all-cause mortality, and the log-rank test was used for initial comparisons. The Kaplan-Meier estimates of the survival curves were plotted against time to follow-up period. These curves helped in identifying non-proportionality patterns in hazard function such as convergence (difference in risk between the groups decreases with time), divergence or crossing of the curves. In addition, a Schoenfeld test for the violation of proportional hazards, which can be used to assess the correlation between scaled residuals and time, was also conducted. Univariable and multivariable Cox proportional hazard analyses were used to evaluate changes of LVEF as a predictor of cardiac event rates and all-cause mortality. Univariable parameters with p<0.05 were included in the multivariable analysis. The proportional hazards assumption for the model was checked by examining log minus-log transformed data. A p<0.05 was considered statistically significant for all comparisons, and all analyses were performed using a statistical software package (SPSS V.24.0).

## Results

The clinical characteristics of patients who underwent the second LVEF assessment are presented in table 1. The rEF group had a lower body mass index and higher heart rate when compared with the pEF group. In addition, the prevalences of male gender, CAD, diabetes, CKD, anaemia, hyperuricaemia and SDB were highest in the rEF group among the groups. The findings of laboratory data, ECG and echocardiography are presented in table 2. BNP, troponin I, total bilirubin, LVDd, LVDs and QRS were highest, and LVEF was lowest in the rEF group. In contrast, other parameters, including C reactive protein, total protein, mitral valve E/E’, IVC, TR-PG, RV-FAC, PQ and QT, did not significantly differ among the groups. In the multivariable logistic regression analysis (table 3), younger age, presence of CAD and SDB, higher levels of troponin I, LVDd, and lower levels of LVEF at the first assessment were predictors of rEF (worsened HFpEF).

**Table 1:** Clinical features of patients with HFpEF at first LVEF assessment, and changes in LVEF observed at second assessment (n=1082)

	pEF (=758)	mrEF (=138)	rEF (=186)	P value
Age (years)	67.8±14.5	67.5±13.2	65.2±15.8	0.086
Male gender (n, %)	404 (53.3)	87 (63.0)	123 (66.1)	0.002
Body mass index (kg/m^2^)	23.5±4.3	23.1±3.8	22.6±3.6*	0.025
Systolic blood pressure (mm Hg)	130.4±30.2	135.2±30.8	130.1±33.3	0.223
Diastolic blood pressure (mm Hg)	72.8±31.2	74.7±24.5	74.8±22.9	0.610
Heart rate (bpm)	75.5±23.8	77.5±24.6**	86.6±25.3**	<0.001
NYHA functional class III/IV (n, %)	26 (3.4)	2 (1.4)	7 (3.8)	0.435
**Comorbidity**				
Coronary artery disease (n, %)	187 (24.7)	44 (31.9)	78 (41.9)	<0.001
Atrial fibrillation (n, %)	290 (38.3)	64 (46.4)	86 (46.2)	0.048
Hypertension (n, %)	521 (68.7)	94 (68.1)	140 (75.3)	0.199
Diabetes (n, %)	248 (32.7)	51 (37.0)	83 (44.8)	0.009
Dyslipidaemia (n, %)	524 (69.1)	113 (81.9)	134 (72.0)	0.009
Chronic kidney disease (n, %)	353 (46.6)	80 (58.0)	109 (58.6)	0.002
Anaemia (*n*, %)	381 (50.3)	82 (59.4)	120 (64.5)	0.001
Hyperurecaemia (n, %)	375 (49.5)	92 (66.7)	135 (72.6)	<0.001
Sleep-disordered breathing (n, %)	253 (33.4)	62 (44.9)	99 (53.2)	<0.001
COPD (n, %)	171 (22.6)	35 (25.4)	51 (27.4)	0.337
Peripheral artery disease (n, %)	67 (8.8)	12 (8.7)	25 (13.4)	0.150
Smoking (n, %)	385 (51.7)	78 (58.2)	95 (51.4)	0.359
Alcohol (n, %)	69 (9.3)	16 (11.9)	12 (6.5)	0.240
**Treatment**				
RAS inhibitor (n, %)	484 (63.9)	97 (70.3)	146 (78.5)	<0.001
Mineral receptor antagonist (n, %)	215 (28.4)	54 (39.1)	101 (54.3)	<0.001
Calcium channel blocker (n, %)	322 (42.5)	54 (39.1)	73 (39.2)	0.604
Beta blocker (n, %)	440 (58.0)	111 (80.4)	155 (83.3)	<0.001
Diuretic (n, %)	415 (54.7)	93 (67.4)	147 (79.0)	<0.001
Statin (n, %)	295 (39.6)	56 (41.8)	75 (40.5)	0.882
Digitalis (n, %)	86 (11.5)	11 (8.2)	21 (11.4)	0.523
Amiodarone (n, %)	55 (7.3)	14 (10.1)	36 (19.4)	<0.001
Antiplatelet agent (n, %)	353 (46.6)	83 (60.1)	114 (61.3)	<0.001
Anticoagulant (n, %)	39 6 (52.2)	95 (68.8)	126 (67.7)	<0.001
PCI (n, %)	123 (16.2)	40 (29.0)	59 (31.7)	<0.001
Catheter ablation (n, %)	82 (10.8)	10 (7.2)	19 (10.2)	0.445
ICD (n, %)	91 (12.2)	16 (11.9)	26 (14.1)	0.778

*P<0.05, **P<0.01 vs pEF, †p<0.05 and ††p<0.01 vs mrEF.

COPD, chronic obstructive pulmonary disease; HFpEF, heart failure with preserved LVEF; ICD, implantable cardiac defibrillator; LVEF, left ventricular ejection fraction; mrEF, mid-range LVEF at second assessment; NYHA, New York Heart Association; PCI, percutaneous coronary intervention; pEF, remained preserved LVEF at second assessment; RAS, renin–angiotensin–aldosterone system; rEF, reduced LVEF at second assessment.

**Table 2:** Laboratory and echocardiographic data of patients with HFpEF at first LVEF assessment, and whose changes in LVEF at second assessment (n=1082)

	pEF (=758)	mrEF (=138)	rEF (=186)	P value
**Laboratory data**				
White cell count (*10^3^/uL)	6.9±3.1	7.3±3.4	7.8±3.7	0.045
Haemoglobin (g/dL)	12.5±2.3	12.5±2.2	12.0±2.2**	0.025
BNP (pg/mL)†	135.2 (53.5–329.6)	194.0 (53.9–520.9)	319.8 (93.3–702.0) **†	<0.001
Troponin I (ng/mL) †	0.040 (0.017–0.080)	0.040 (0.017–0.078)	0.069 (0.032–0.355)*†	0.033
eGFR (mL/min/1.73 cm^2^)	60.1±23.3	55.2±24.3	55.7±25.2	0.042
C reactive protein (mg/dL) †	0.13 (0.05–0.48)	0.18 (0.05–1.35)	0.38 (0.09–1.08)	0.530
Total protein (g/dL)	6.9±0.7	6.9±0.8	6.9±0.7	0.542
Albumin (g/dL)	3.8±0.6	3.7±0.6	3.7±0.5	0.072
Total bilirubin (mg/dL)	0.8±0.4	0.9±0.5	1.0±0.57**†	<0.001
Direct bilirubin (mg/dL)	0.1±0.1	0.1±0.1	0.1±0.1	0.771
Sodium (mEq/L)	139.0±3.4	138.5±3.8	138.0±3.5†	0.001
**Echocardiographic data**				
LVEF (%)	62.9±7.2	60.6±9.6**	55.1±11.6**†	<0.001
LVDd (mm)	45.6±8.3	49.7±9.1**	53.2±10.9**††	<0.001
LVDs (mm)	29.2±8.2	33.6±10.1**†	39.2±12.5**†	<0.001
Left atrium volume (mL)	67.8±44.7	86.6±68.5**	84.2±69.9*††	<0.001
Mitral valve E/E’	13.0±7.6	14.9±9.8	13.2±7.5	0.108
IVC (mm)	14.4±4.7	14.7±5.2	14.6±4.4	0.637
TR-PG (mm Hg)	31.5±18.7	30.7±17.5	29.2±15.2	0.440
RV-FAC (%)	42.6±13.9	43.9±14.9	41.9±13.5	0.659
**ECG**				
Rhythm sinus/atrial fibrillation/pacing (n, %)	554 (73.1)/127 (16.8)/77 (10.2)	93 (67.4)/30 (21.7)/15 (10.9)	117 (62.9)/41 (22.0)/28 (15.1)	0.062
CRBBB (n, %)	77 (10.2)	10 (7.2)	17 (9.1)	0.550
CLBBB (n, %)	5 (0.7)	2 (1.4)	4 (2.2)	0.166
Heart rate (excluding pacing, n=465)	70.4±15.6	69.2±13.1**	75.0±15.1**	<0.001
PQ (ms)	175.3±35.7	176.8±40.2	182.0±44.8	0.214
QRS (ms)	106.9±20.7	108.1±22.7	112.3±25.5*†	0.036
QT (ms)	410.6±45.1	415.2±49.4	409.0±47.4	0.455
QTc (ms)	441.3±34.3	445.4±37.2	451.7±35.8**	0.002

*P<0.05, **P<0.01 vs pEF, †p<0.05 and ††p<0.01 vs mrEF.

†Data are presented as median (IQR).

BNP, B-type natriuretic peptide; CLBBB, complete left bundle branch block; CRBBB, complete right bundle branch block; GFR, glomerular filtration rate; HFpEF, heart failure with preserved LVEF; IVC, inferior vena cava diameter; LVDd, left ventricular end diastolic dimension; LVDs, left ventricular end systolic dimension; LVEF, left ventricular ejection fraction; mrEF, mid-range LVEF at second assessment; pEF, remained preserved LVEF at second assessment; rEF, reduced LVEF at second assessment; RV-FAC, right ventricular fractional area change; TR-PG, tricuspid regurgitation pressure gradient.

**Table 3:** Logistic regression analysis: associations between the clinical profiles and ‘rEF at second LVEF assessment’

	Univariable			Multivariable		
	OR	95% CI	P value	OR	95% CI	P value
Age	0.988	0.978 to 0.999	0.028	0.974	0.953 to 0.995	0.016
Male gender	1.610	1.157 to 2.242	0.005	0.998	0.505 to 1.971	0.995
Body mass index	0.948	0.909 to 0.988	0.012	0.933	0.865 to 1.007	0.073
Systolic blood pressure	0.999	0.994 to 1.004	0.666			
Heart rate	1.016	1.010 to 1.022	<0.001	1.012	0.996 to 1.029	0.138
NYHA class III or IV	1.212	0.521 to 2.819	0.655			
Coronary artery disease	2.079	1.499 to 2.885	<0.001	2.112	1.047 to 4.259	0.037
Atrial fibrillation	1.317	0.958 to 1.809	0.090			
Hypertension	1.391	0.968 to 1.997	0.074			
Diabetes	1.609	1.168 to 2.217	0.004	1.517	0.789 to 2.915	0.211
Dyslipidaemia	1.048	0.739 to 1.489	0.795			
Chronic kidney disease	1.514	1.099 to 2.084	0.011	0.694	0.342 to 1.409	0.312
Anaemia	1.700	1.225 to 2.360	0.001	1.378	0.699 to 2.719	0.355
Hyperuricaemia	2.432	1.717 to 3.443	<0.001	1.372	0.681 to 2.762	0.376
Sleep-disordered breathing	2.099	1.526 to 2.888	<0.001	1.212	1.007 to 1.472	0.042
COPD	1.265	0.885 to 1.810	0.197			
Peripheral artery disease	1.606	0.993 to 2.596	0.093			
Smoking	0.948	0.691 to 1.302	0.743			
Alcohol	0.648	0.346 to 1.212	0.175			
Log BNP	2.600	1.864 to 3.628	<0.001	1.543	0.845 to 2.817	0.158
Log troponin I	1.465	1.222 to 1.756	<0.001	1.574	1.055 to 2.349	0.026
LVDd	1.080	1.059 to 1.101	<0.001	1.069	1.030 to 1.109	<0.001
LVEF	0.908	0.888 to 0.927	<0.001	0.933	0.902 to 0.966	<0.001
CLBBB	2.791	0.809 to 9.634	0.104			
QRS	1.008	1.001 to 1.015	0.026	0.997	0.983 to 1.011	0.662
QT	0.999	0.995 to 1.003	0.559			
QTc	1.008	1.003 to 1.012	0.002	1.004	0.995 to 1.013	0.409
RAS inhibitors	0.725	0.547 to 0.936	0.037	0.931	0.472 to 1.388	0.281
Mineral receptor antagonist	0.926	0.562 to 1.324	0.463			
Calcium channel blocker	0.893	0.647 to 1.234	0.494			
Beta blocker	0.892	0.413 to 1.326	0.782			
Diuretic	2.879	1.975 to 4.197	<0.001	2.272	0.970 to 5.320	0.414
Statin	1.297	0.943 to 1.783	0.110			
Digitalis	1.501	0.915 to 2.462	0.108			
ICD	1.180	0.744 to 1.871	0.482			

BNP, B-type natriuretic peptide; CLBBB, complete left bundle branch block; COPD, chronic obstructive pulmonary disease; HFpEF, heart failure with preserved LVEF; ICD, implantable cardiac defibrillator; LVDd, left ventricular end diastolic dimension; LVEF, left ventricular ejection fraction; LVEF, left ventricular ejection fraction; NYHA, New York Heart Association; RAS, renin–angiotensin–aldosterone system; rEF, reduced LVEF at second assessment.

**Figure 1:**
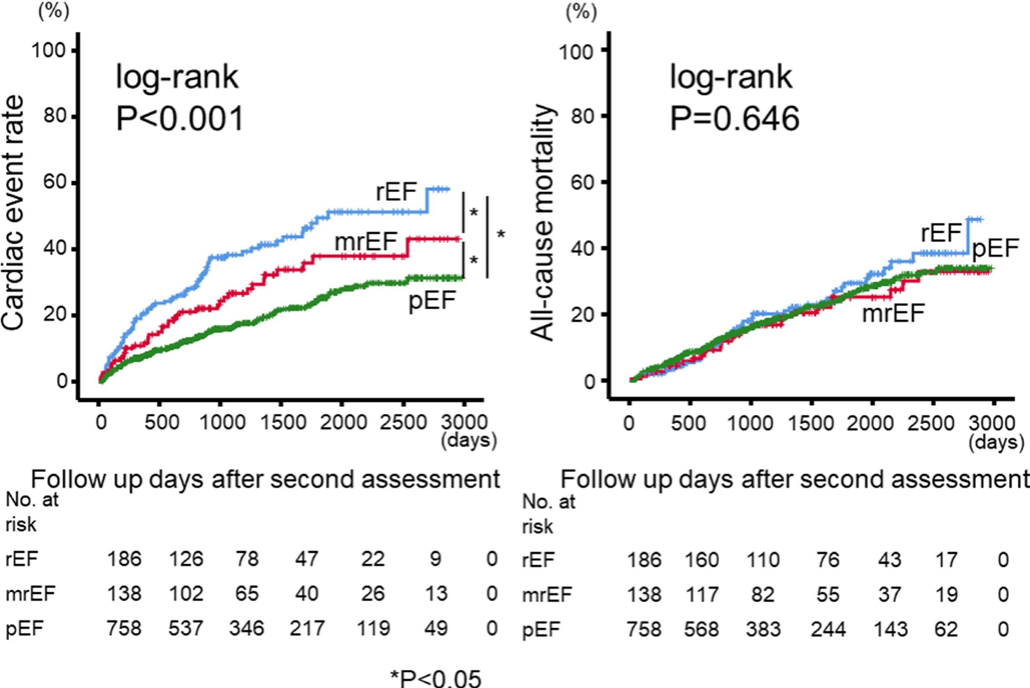
Rates of cardiac events and all-cause mortality with changes in left ventricular ejection fraction (LVEF) in heart failure patients with preserved LVEF (HFpEF). Kaplan-Meier analysis, during the follow-up period after the second assessment of LVEF, for cardiac event rate and all-cause mortality based on changes in LVEF between the first and second assessments. remained preserved LVEF (pEF) (LVEF ≥50%, n=758); mid-range LVEF (mrEF) (LVEF 40%–49%, n=138); and reduced LVEF (rEF) (LVEF <50%, n=186) at the second assessment.

During the follow-up period (mean 1228±790 days, range 15–2975 days), 252 cardiac events, including 218 hospitalisations due to HF and 34 cardiac deaths, occurred, as well as 226 all-cause mortalities (91 cardiac deaths and 135 non-cardiac deaths). In the Kaplan-Meier analysis (figure 1), the cardiac event rate of the groups progressively increased from pEF, mrEF to rEF (log-rank, p<0.001), whereas all-cause mortality did not significantly differ among the groups. In the univariable and multivariable Cox proportional hazard analyses (table 4), rEF (vs pEF) was not a predictor of all-cause mortality, but an independent predictor of increased cardiac event rates after adjustment of LVEF at the first assessment (HR 1.424, 95% CI 1.020 to 1.861, p=0.039).

**Table 4:** Cox proportional hazard model of cardiac events and all-cause mortality in HFpEF

	HR	95% CI	P value
**Cardiac event (252 events/1082 patients**)			
pEF	Ref		
mrEF	1.593	1.119 to 2.266	0.010
mrEF adjusted*	1.185	0.728 to 1.732	0.543
rEF ‘worsened HFpEF’	2.439	1.842 to 3.230	<0.001
rEF ‘worsened HFpEF’ adjusted*	1.424	1.020 to 1.861	0.039
**All-cause mortality (226 events/1082 patients**)			
pEF	Ref		
mrEF	0.925	0.621 to 1.377	0.699
rEF ‘worsened HFpEF’	1.134	0.819 to 1.570	0.450

*Adjusted: adjusted for age, gender, body mass index, systolic blood pressure, heart rate, New York Heart Association class III or IV, presence of coronary artery disease, atrial fibrillation, hypertension, diabetes, dyslipidaemia, chronic kidney disease, anaemia, hyperuricaemia, sleep-disordered breathing, chronic obstructive pulmonary disease, smoking, alcohol, usage of renin–angiotensin–aldosterone system inhibitors, mineral receptor antagonist, calcium channel blocker, beta blockers, diuretics, statin, digitalis, implantable cardiac defibrillator, B-type natriuretic peptide, tricuspid regurgitation pressure gradient, right ventricular fractional area change, mitral regurgitation, left atrium volume and left ventricular ejection fraction at first assessment.

HFpEF, heart failure with preserved LVEF; mrEF, mid-range LVEF at second assessment; pEF, remained preserved LVEF at second assessment; rEF, reduced LVEF at second assessment.

## Discussion

In the present study, we demonstrated that patients with rEF; worsened HFpEF, which was 17.2% in the present study, were associated with younger age, higher presence of several comorbidities, including CAD and SDB, and higher levels of troponin I and LVDd, indicating the presence of myocardial damage and structural remodelling, and worse cardiac event rate. However, all-cause mortality did not necessarily differ with LVEF changes in the HFpEF patients.

It has been reported that there were differences in prognostic factors between HFrEF and HFpEF.^27 28^ Older age and diabetes mellitus were predictors of HFrEF and HFpEF, and higher body mass index and AF were predictors of HFpEF, whereas male gender, higher heart rate, hypertension, cardiovascular disease, left ventricular hypertrophy and left bundle-branch block were predictors of HFrEF risk.^29 30^

Although LVEF assessment is generally used to predict the prognosis and select the treatment for HF,^1–5^ LVEF changes and their prognostic impacts on HFrEF, have recently been reported as ‘recovered EF’.^8–14^ However, there are few reports on the prognostic impact of LVEF changes in HFpEF patients. In the present study, worsened HFpEF was not a predictor of all-cause mortality, but an independent predictor of increased cardiac event rates after adjustment for baseline LVEF. Thus, not only baseline LVEF, but also its changes, seem to be associated with cardiac event rates in HFpEF patients. Tsuji *et al* reported that worsened HFpEF was observed in only 1.9% of stable HFpEF patients over a 1-year period, and was associated with higher all-cause mortality compared with patients with persistent HFpEF.^31^ Dunlay *et al* reported that EF progressively decreases with ageing in HF patients, and that a decrease in LVEF was associated with prevalence of CAD, as well as reduced survival.^32^

Male gender,^8^ CAD,^6 33^ AF,^34^ diabetes,^6 33 35^ CKD,^6 33 35^ anaemia,^33 35^ hyperuricaemia^19^ and SDB^35–37^ have been reported to be associated with left ventricular remodelling and adverse prognosis in HF patients. However, younger age, non-ischaemic aetiology and fewer comorbidities are associated with left ventricular reverse remodelling in HF patients.^2^ In particular, compared with HFrEF, HFpEF has many comorbidities, which contribute to HF progression.^1 6^ LVEF itself is not necessarily associated with mortality, and non-cardiac comorbidity has a greater prognostic impact on HFpEF than HFrEF.^28 38^ Concordant with these findings,^28 38^ in the present study, non-cardiac mortality was higher than cardiac mortality in HFpEF patients.

### Study strengths and limitations

There are several strengths to our study. This is the first study to show changes in LVEF, comprehensive confounding factors for changes in LVEF and their prognostic impacts in HFpEF patients.

The present study also has several limitations. First, as a prospective cohort study of a single centre with a relatively small number of patients, the present results may not be representative of the general population. Second, we could not examine all patients, who had undergone the first assessment LVEF, at the second assessment (93.2%) because of losing follow-up and/or occurrence of event before the second assessment, and selection bias could not be fully denied. Although LVEF was reassessed in the outpatient setting within half a year, the time periods between the first and second assessments differ from patient to patient. Third, the present study included only variables relating to hospitalisation for decompensated HF, and we did not take into consideration changes in medical parameters or treatments, other than LVEF. Therefore, the present results should be viewed as preliminary, and further studies with larger populations are needed.

## Conclusions

An initial assessment of LVEF and LVEF changes are important for deciding treatment and predicting prognosis in HFpEF patients. In addition, several confounding factors are associated with LVEF changes in worsened HFpEF patients.
